# Insights into the interactions and dynamics of a DES formed by phenyl propionic acid and choline chloride

**DOI:** 10.1038/s41598-021-85260-z

**Published:** 2021-03-18

**Authors:** Parisa Jahanbakhsh Bonab, Alireza Rastkar Ebrahimzadeh, Jaber Jahanbin Sardroodi

**Affiliations:** 1 Department of Chemistry, Faculty of Basic Sciences, Azarbaijan Shahid Madani University, Tabriz, Iran; 2 Molecular Simulation Laboratory (MSL), Azarbaijan Shahid Madani University, Tabriz, Iran; 3 Computational Nanomaterials Research Group (CNRG), Department of Physics, Faculty of Basic Sciences, Azarbaijan Shahid Madani University, Tabriz, Iran

**Keywords:** Chemical physics, Thermodynamics

## Abstract

Deep eutectic solvents (DESs) have received much attention in modern green chemistry as inexpensive and easy to handle analogous ionic liquids. This work employed molecular dynamics techniques to investigate the structure and dynamics of a DES system composed of choline chloride and phenyl propionic acid as a hydrogen bond donor and acceptor, respectively. Dynamical parameters such as mean square displacement, liquid phase self-diffusion coefficient and viscosity are calculated at the pressure of 0.1 MPa and temperatures 293, 321 and 400 K. The system size effect on the self-diffusion coefficient of DES species was also examined. Structural parameters such as liquid phase densities, hydrogen bonds, molecular dipole moment of species, and radial and spatial distribution functions (RDF and SDF) were investigated. The viscosity of the studied system was compared with the experimental values recently reported in the literature. A good agreement was observed between simulated and experimental values. The electrostatic and van der Waals nonbonding interaction energies between species were also evaluated and interpreted in terms of temperature. These investigations could play a vital role in the future development of these designer solvents.

## Introduction

In the last decades, a large number of studies have been conducted to replace organic solvents with new eco-friendly ones. These approaches comprise the use of easy recyclable systems, such as supercritical CO_2_ or fluorous solvents, ionic liquids (ILs), deep eutectic solvents (DESs), and low melting mixtures (LMMs)^1^. Ionic liquid solvents have many unique physicochemical properties, such as low vapor pressures, non-flammability, wide liquid range, high thermal conductivity and stability. However, most ILs also have some disadvantages such as toxicity, poor biodegradability, and very low tolerance to moisture, thus limiting their industrial usage^2–6^. Abbot et al.^7^ introduced DES in 2001, stating that they could be classified as a new type of ILs. The physicochemical properties of DESs, such as density, viscosity, conductivity and surface tension are very close to those of ILs^5,6^. Unlike ILs, DESs are composed of an ionic mixture rather than a single ionic compound^8^. DESs are a mixture of two or more non-toxic and cheap components, namely, hydrogen bond acceptor (HBA) and hydrogen bond donor (HBD), which have a lower melting point when compared to the pure components^8,9^. HBD acts as a complexing agent strongly interacting with the anion, thus causing the increase of its effective size. Subsequently, the interaction of anions with cations could be decreased, providing a reasonable explanation for the depression of the melting point of DESs^9–11^. Another reason for the huge depression of the melting point of DESs is the delocalization of the charge throughout the hydrogen bond between HBD and HBA^9,12^. Besides, DESs are much easy to prepare, inexpensive, biodegradable and stable in air; thus, they can be more interesting than ionic liquids for industrialization^13^, with many applications in various areas of chemistry such as electrochemistry^14–16^, organic reactions^6,17^, and enzyme reactions^18^. DESs have also been utilized in the extraction or separation of gas^19–22^, aromatics hydrocarbons^15^, biodiesels, and bioactive components^23^.

Given the above-mentioned reasons, DESs form a noteworthy and valuable class of solvents in chemistry^9,13,24,25^. Therefore, it is essential to investigate their structural and physicochemical properties at different temperatures to develop the knowledge necessary for the optimal design of DESs for chemical processes. Also, specifying the composition of DESs is hard when predicted by trial and error^24,26,27^. If the dominant interactions in DESs are determined, the eutectic point may be anticipated. Molecular dynamics (MD) simulations offer reliable approaches to investigate the structural and physicochemical properties and molecular interactions of DESs.

The hydrogen- bond plays a vital role in determining the physicochemical properties of DESs. Density Functional Theory (DFT) method was applied by Ashworth^28^ to evaluate the role of hydrogen bonds in DESs. Additionally, the relationship between the variation of electron density at the cage critical points of hydrogen bond networks and melting point for a set of 45 choline chloride-based DESs was investigated and rationalized with DFT studies combined with a topological analysis of electron density^29^. Sun et al.^30^ also considered a mixture of choline chloride and urea to evaluate the structures of these systems with different urea concentrations using MD simulation methods. Colina’s group^31,32^ explored the interactions of species in choline chloride-based DES by MD simulations and experimental techniques to beter understand their behavior. Briesen and co-workers^27^, on the other hand, studied four deep eutectic solvents by MD simulation using two force fields, aiming to investigate the force field’s effect on inaccurate results. Besides, Celebi et al.^33^ performed MD simulations to predict molecular interactions, microscopic structure and the thermophysical properties of aqueous choline chloride-based DESs solutions. They intended to find how the addition of water on DESs could affect the structural and dynamical properties of them.

In chemical productions, acid-catalyzed reactions serve an essential role; so extensive studies have been performed on acidic ionic liquids (AILs)^34^. Therefore, similar to ionic liquids, it is necessary to investigate acidic deep eutectic solvents (ADESs) considering their unique properties. More importantly, ADESs, which have a freezing point lower than 50 °C, should be considered^35^. For the first time, Abbott et al.^36^ investigated the properties of an ADES composed of carboxylic acids and choline chloride experimentally; they also characterized phase behavior, fluidity and conductivity as a function of composition and acid type. This ADES has wide application in dissolution and extraction. So, it is crucial to focuse on and understand the behavior of ADES systems industrial applications^32,33^. Likewise, for the optimal design of ADES for a specific chemical process, it is necessary to obtain more information regarding the structural, physicochemical and transport properties of ADESs, as well as their relationship with temperature, thus helping to accurately design a task-specific solvent. Also, developing ADES-based technologies requires finding the most suitable molecular interactions, and structural and dynamical properties at different temperatures.

**Figure 1:**
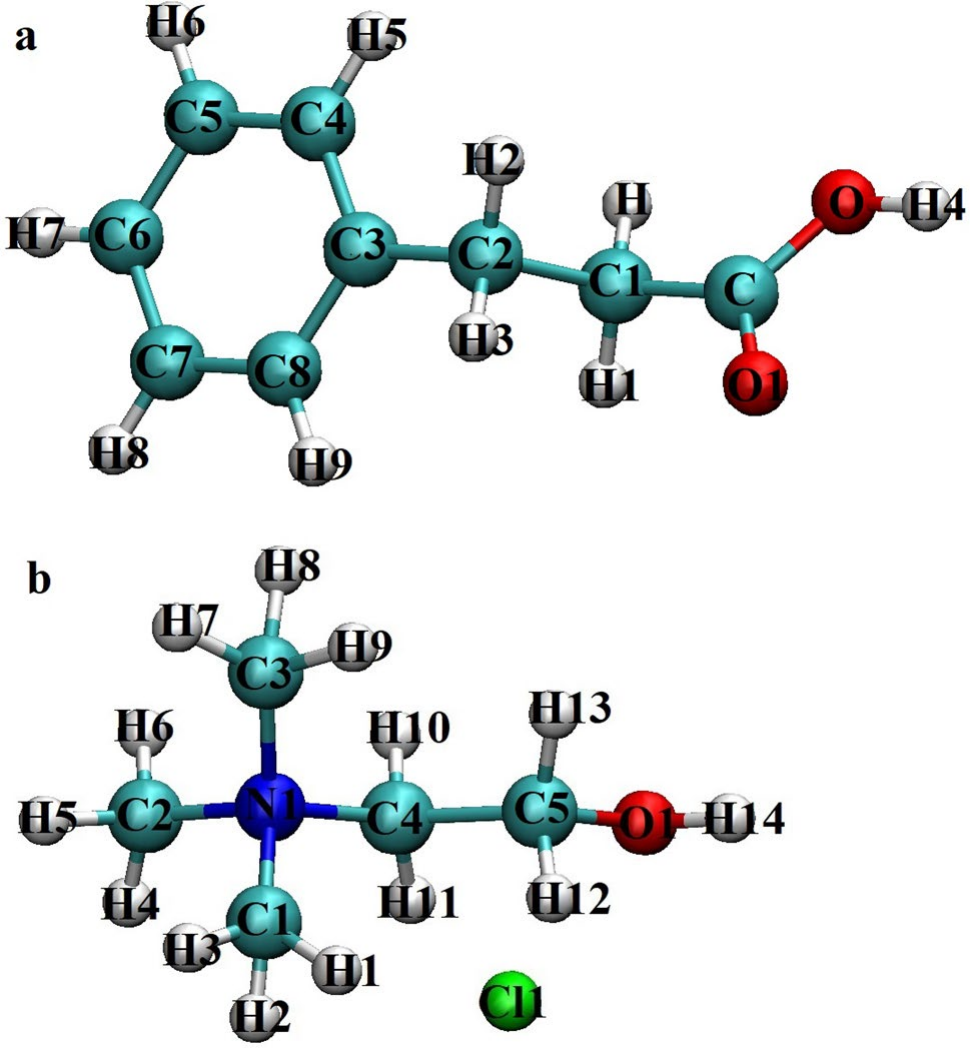
Structures and atom type notations of (**a**) phenyl propionic acid, Phpr, (**b**) choline chloride, ChCl. Atom color code: (Cyan) carbon, (red) oxygen, (blue) nitrogen, (green) chloride ion, (light gray) hydrogen. Atomic label was used in molecular dynamics simulations. The molecular structures were visualized by the VMD package^40^.

In this work, the effects of temperature on the dynamical and structural properties of an ADES composed of phenyl propionic acids (Phpr) as HBD and choline chloride (ChCl) as HBA with 67 mol% Phpr were investigated through MD simulations. After determining the structural and dynamical properties of this ADES, the capability of this ADES in the sweetening of natural gas will be investigated in our future studies. The structure of the components of the DES studied in this paper is shown in Fig. 1.

## Results and discussion

### Radial distribution function

To qualify the micro-structure of the DESs, a structural analysis in the form of radial distribution functions (RDF) was conducted to get more information at the molecular level. The eutectic point of the DES studied in this paper, as formed at a composition of 67 mol% Phpr, was at the temperature of 293 K^36^.

**Figure 2:**
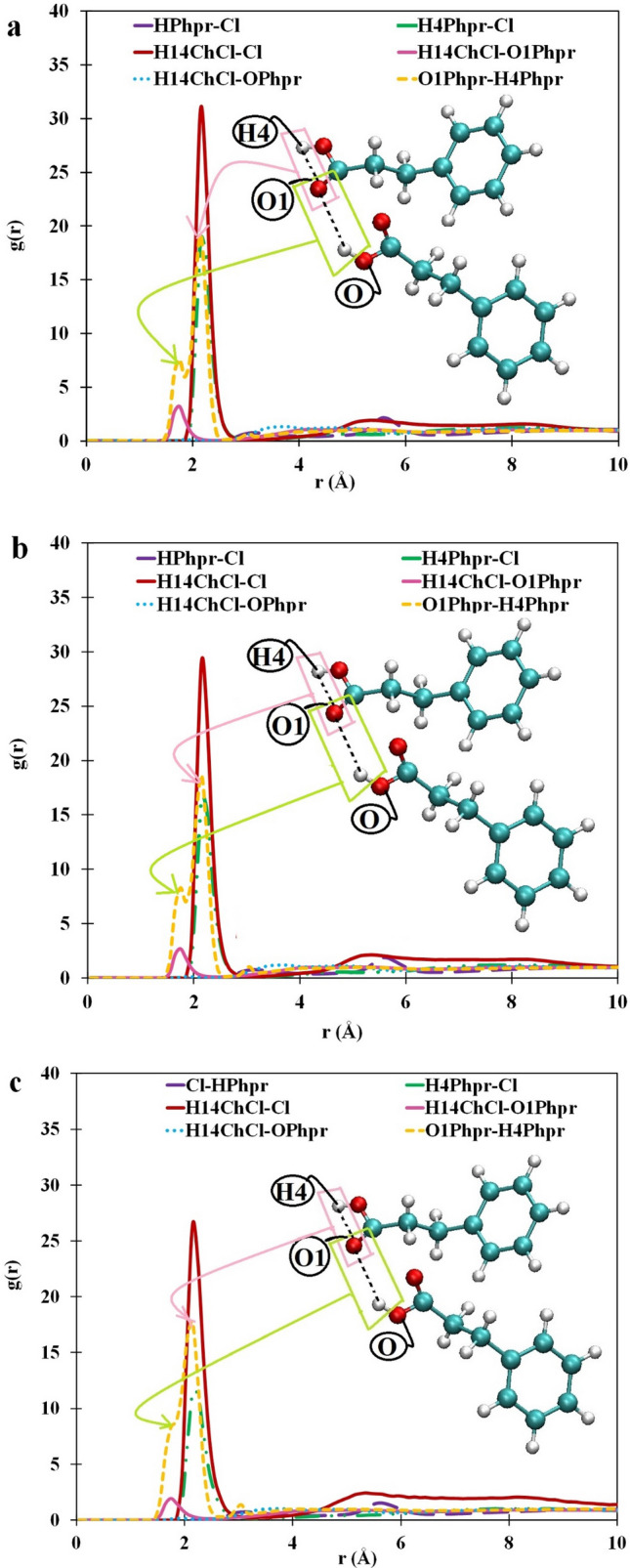
Atom–atom RDFs for Phpr–Phpr (yellow), Phpr-choline (blue and pink), Phpr–Cl^−^ (green, violet), and choline-Cl^−^ (red) at (**a**) 293 K, (**b**) 321 K and (**c**) 400 K were obtained using the VMD package^40^.

We considered the choline cation and Phpr molecules as the reference for the atom–atom RDFs, as illustrated in Fig. 2. The probability distributions of the hydroxyl hydrogen atom of Phpr (H4) and chloride anion around Phpr molecules were obtained. As can be seen in Fig. 2, there were three intensive peaks at a short distance, which were related to the Cl^−^–H14 of choline cation, Cl^−^–H4 of Phpr, and O1 of Phpr–H4 of Phpr (O1–H4) RDFs. The RDF’s peaks for the chloride anion around Phpr through the H4 site were stronger than those for the H14 of the choline cation around O1 and O of Phpr. The RDF of the choline cation at the site of hydroxyl hydrogen (H14) was investigated around the two possible acceptor sites in Phpr (O and O1), showing that the first maximum peak of the choline cation with Phpr molecule through the O1 site (H14–O1) appeared at nearly 1.65 Å shorter than the distance of the first maximum peaks of the Cl^–^–H4 of Phpr in the studied systems. Moreover, the RDF of the H14 of choline around O1 of Phpr was located almost at the same position as O1 of the Phpr–H4 of the Phpr’s RDF; thus, they might have identical interaction types. Therefore, it could be concluded that the first peak of the O1–H4’s RDF was related to the intermolecular interactions. It should also be noted that Phpr has two sites that may act as the hydrogen bond acceptor and donor: (1) the oxygen atom in –C=O for the carboxylate group (O1) as the acceptor and (2) –OH in the –COO group (O) as the donor. The O1–H4’s RDF has two peaks at 1.7 Å and 2.18 Å, being near each other. This phenomenon indicates that this interaction serves a vital role in the formation of the hydrogen bonds network. The first peak is related to the intermolecular interaction of Phpr–Phpr; in other words, one Phpr molecule acts as an acceptor; the other ones serves as hydrogen bonds donor. The second peak position of O1–H4 was found at 2.18 Å, with an intensive frequency, mainly owing to the intramolecular interactions of the Phpr molecules between H4 and O1.

As can be seen in Fig. 2, the wide peak that appeared at roughly 3.85 Å was related to the weak interaction between the O of the Phpr site and the H14 of ChCl. The narrow and robust peak at the short distance indicated a strong interaction, whereas insignificant features became visible at long distances. Therefore, the interaction of choline-Phpr was mainly related to the O1 site in all considered systems since the first maximum peak of H14-O1 appeared in the short distance.

We also characterized the RDF between anion (Cl^−^) and hydrogen atoms covalently bonded to carbon atoms (H/H1). They served no significant role in the formation of the hydrogen bond network since the first maximum peak appeared in the long distance with an insignificant intensity in all studied DES.

The effect of temperature on the DESs’ RDF was also been investigated. Our findings, as reported in Fig. 2a–c, indicated that the intensity of the peaks was decreased with the rise of temperature. The reason for this was that as the temperature was raised, the average distance between molecules was increased, and the intermolecular interaction was weakened. The position of the maximum peaks of the RDFs was partially shifted to the right as the temperature was increased. At the eutectic temperature, 293 K, the intensity of Phpr–Cl^−^ and Phpr–Phpr RDFs was identical, but the rise of temperature led to a significant difference in the intensity of the peaks obtained. In other words, the chloride anions did not tend to be located near the choline cation at high temperatures.

The coordination number (N_C(i−j)_) is calculated by the integration of the first maximum peak of RDFs and the absolute number of the first neighbors, as listed in Table 1. These results represented the maximum coordination number in the studied DESs,which was related to the hydroxyl hydrogen of Phpr (H4) when located around the other Phpr molecule and chloride anions; in other words, there were two Phpr molecules and two chloride anions around the Phpr molecule in the first solvation of the shell, which was in a good agreement with the experimental results obtained by the Abbot group^36^.

**Table 1:** Coordination number (N_C(i−j)_) of Phpr, choline and chloride iones obtained by molecular dynamics simulations.

N_C(i-j)_	First min position (Å)	Coordination number at:		
		293 K	321 K	400 K
N_C (O1–H14)_	2.05	0.120	0.100	0.077
N_C (O–H14)_	2.35	0.008	0.010	0.012
N_C (Cl–H14)_	3.05	0.87	0.86	0.87
N_C(H4–Cl)_	3.05	1.28	1.18	0.51
N_C(H–Cl)_	4.25	0.400	0.386	0.175
N_C (O1–H4)_	2.55	1.265	1.285	1.296

N_C(i−j)_ represents the number of ith molecules around the jth molecule at the first minimum position of RDFs.

### Spatial distribution function

**Figure 3:**
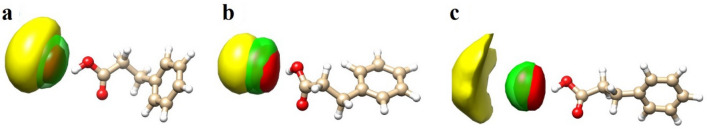
Spatial distribution functions of relevant atoms around phenyl propionic acid in DES at (**a**) 298 K, (**b**) 321 K, (**c**) 400 K and 0.1 MPa were calculated using TRAVIS^61^ software: yellow, choline cation; green, chloride anion; red, Phpr molecule. The isodensity used for the SDF corresponding to choline cation, Phpr, and chloride anion are 2, 2, and 4 times bulk density.

The spatial distribution function analyses, SDFs, were carried out to obtain detailed information on the molecular structure of the eutectic mixture of Phpr and ChCl in 67 mol% Phpr at 293, 321 and 400 K. The SDFs indicated the average density distribution of the chloride anion, choline cation, and Phpr around a Phpr molecule as a reference in the DESs, as shown in Fig. 3. The Phpr–Cl^−^ interactions, as demonstrated in Fig. 3 (green cap), indicated the high-density cap above the hydroxyl group’s hydrogen atom. This confirmed that the sharp peak appearing in 2.14 Å was related to H4–Cl’s RDF. The SDFs for the hydroxyl hydrogen atom (H14) in the choline cation around the Phpr molecule are depicted in Fig. 3 (yellow cap), showing the low-density cap was located above the oxygen atom in the carboxyl group of Phpr (O1 and O); this was consistent with the weak peak of the relevant RDFs.

As shown in Fig. 3, the active site of the Phpr molecule (H4) was surrounded by the chloride anion (green cap). Therefore, the highly distributed caps above the hydrogen atom of the carboxyl group in Phpr at a short distance confirmed the hydrogen bond development in this position. Besides, with the rise of temperature, the SDF of the Phpr molecule, chloride anion and choline cation around the Phpr molecule which was far from the vicinity of hydroxyl hydrogen atoms became smaller.

### Hydrogen bond analysis

Hydrogen bond analysis indicates the number of hydrogen bonds (H-bond) formed between species of the studied system. The following criteria are used to identify the hydrogen bonds^37,38^:

1. The cutoff distance between hydrogen donor atoms (D) and the hydrogen acceptor atom (A) is 3.5 Å.
2. An angle cutoff for ∠DHA of 150° is used, where H represents the hydrogen atoms.

The number of H-bonds between various species types was calculated using the obtained results of the RDFs descibed above, implying the possible relationship between atoms that could serve an important role in the H-bond network. Thus, the average number of the H-bonds of H4–Cl^−^, H14–Cl^−^, O1–H4, and H14–O1, which were related to the interactions of Phpr-anion, cation–anion, Phpr–Phpr and cation–Phpr, respectively, was calculated.

The average number of H-bonds was been obtained by fitting the following well-known Gaussian distribution^39^ to the distribution of the number of H-bonds, by applying the Gnuplot-5.2.6 fitting tool (for the obtained results, see Table 2).

**Table 2:** The average value of the number of hydrogen bonds (N_H(i−j)_) in DESs at 293, 321 and 400 K.

Average number of hydrogen bonds	Temperatures (K)		
	293 K	321 K	400 K
N_H(Cl–H4)_	179.37 ± 0.03	176.46 ± 0.1	132.52 ± 0.3
N_H(Cl–H14)_	140.89 ± 0.5	140.04 ± 0.4	137.42 ± 0.2
N_H(O1Phpr–H4Phpr)_	519.08 ± 0.08	529.88 ± 0.2	552.80 ± 1
N_H(O1Phpr-H14ChCl)_	32.76 ± 0.01	32.72 ± 0.1	20.38 ± 0.1

N_H(i−j)_ refers to the number of H-bond formed between molecule i and j.

1 $$\mathrm{F}\left(\mathrm{x}\right)=\frac{\mathrm{a}}{\upsigma .\sqrt{2\uppi }}\mathrm{exp}\frac{-{\left(\mathrm{x}- \stackrel{-}{\mathrm{x}}\right)}^{2}}{2 \cdot {\upsigma }^{2}}$$

In this equation, a, $\upsigma$ and $\stackrel{-}{\mathrm{x}}$ are the adjustable parameter, standard deviation and the average number of H-bond, respectively.

**Figure 4:**
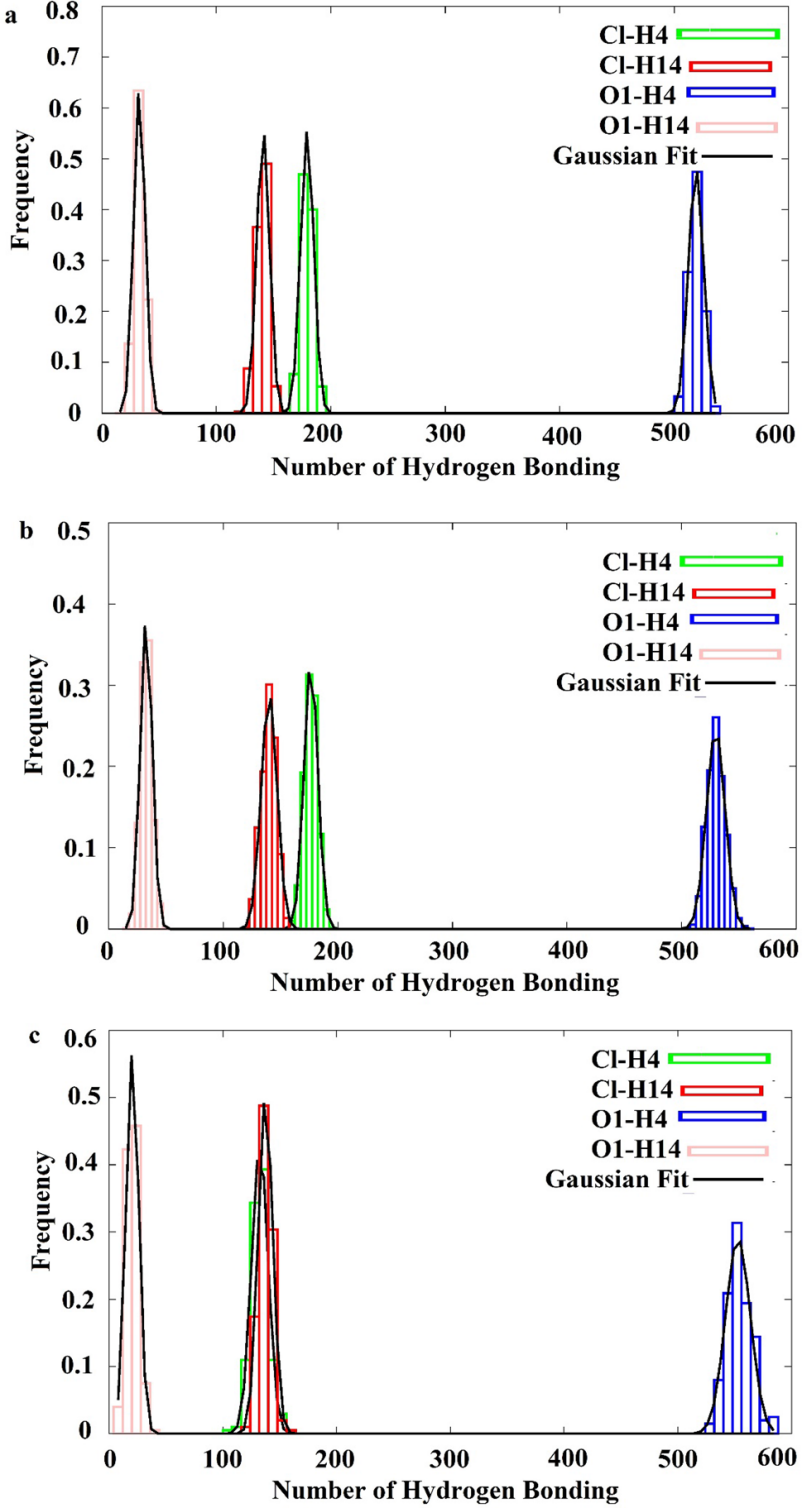
The normalized-distribution of average number of hydrogen bond between species at (**a**) 293 K, (**b**) 321 K and (**c**) 400 K were prepared using Gnuplot 5.2.6 softwre ((http://www.gnuplot.info/). The data were fitted using Gnuplot 5.2.6 (http://www.gnuplot.info/).

Figure 4 displays the normalized distribution of the number of H-bonds for a considered hydrogen bond type at 293, 321 and 400 K. The results suggested that the average number of H-bonds related to O1–H4 (Phpr–Phpr interactions) was more significant than developed between other species. In all considered systems, a relatively large number of H-bonds was developed with Cl^−^ both through the Phpr’ OH group (H4) and the choline’ OH group (H14). The considerable value for H4–Cl^−^ implied that the tendency of Cl^−^ around the choline cation was reduced when the complex of Phpr and anion was formed. This, in turn, contributed to the depression of the melting point of DESs. Besides, the average number of H-bonds developed with the chloride anion through both hydroxyl hydrogen of choline (H14 sites) and carboxyl hydrogen of Phpr (H4 sites) was more significant than that between Phpr through O1 and H14 site of choline in all considered systems.

As shown in Fig. 4, the average number of H-bonds between considered species was decreased, as the temperature of the DESs was increased, except for O1–H4 (Phpr–Phpr interaction). Also, the distribution of the average number of H-bond between Cl^−^ and H4 was similar to developed with Cl^−^ through H14 at 400 K.

**Figure 5:**
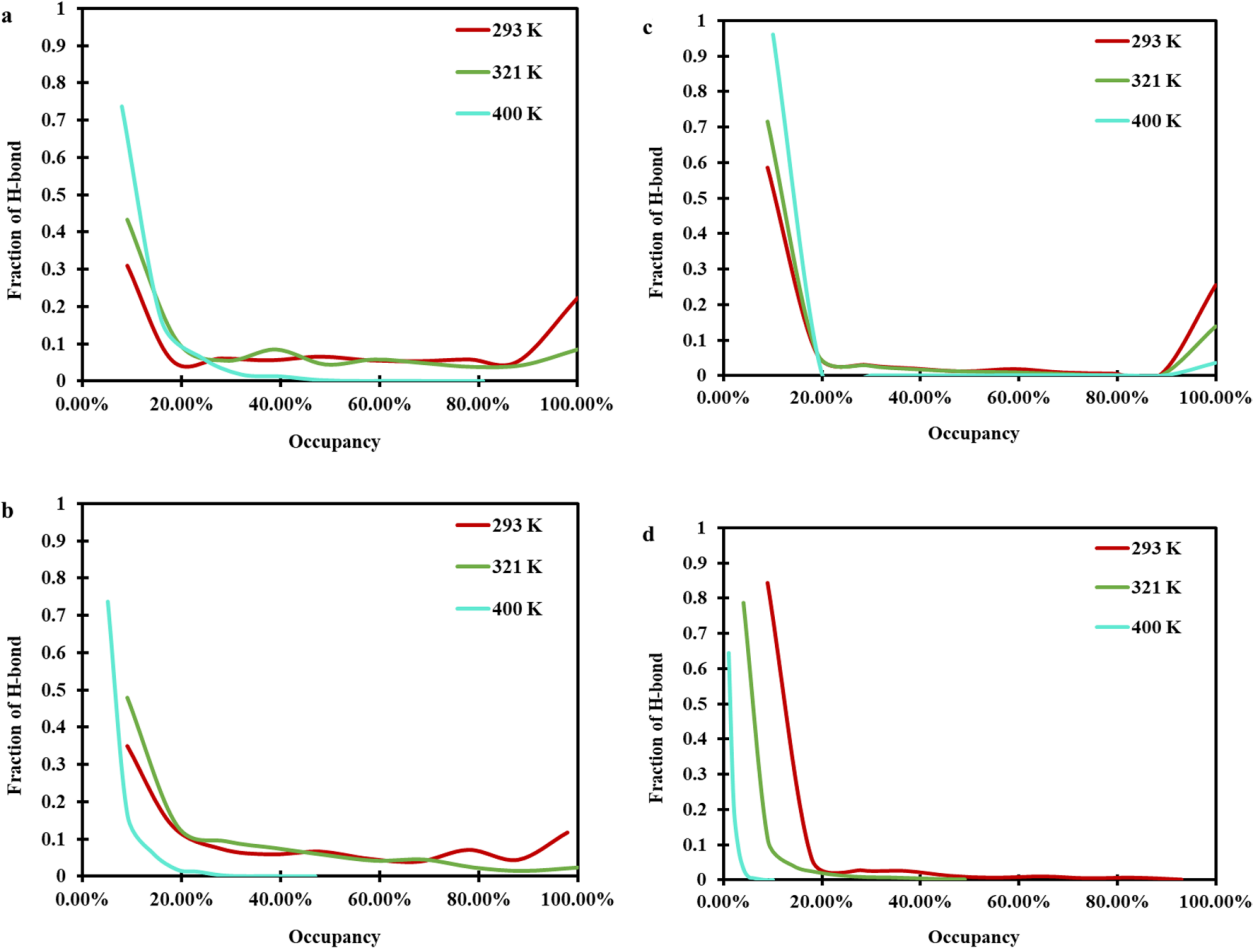
Fraction of hydrogen bond percent occupancies for (**a**) Cl^–^–Phpr (Cl-H4), (**b**) Cl^–^–choline (Cl–H14), (**c**) Phpr–Phpr (O1–H4), and (**d**) Phpr–choline (O1–H14) in DES at 293, 321, and 400 K and 0.1 MPa. The data were analyzed using the VMD package^40^.

Here, we have also reported the percent occupancy of hydrogen bonds, which is described as the total time a unique H-bonds is present during the analyzed trajectory. As can be evidently seen in Fig. 5, the Phpr-Cl^−^ H-bond type had a larger number of H-bonds with a greater percent occupancies in comparision to the other H-bond type (like choline-anion, choline-Phpr); meawhile, this was lower than the Phpr–Phpr’s H-bond. Our results, therefore, illustrated that the most massive occupancy was related to the H-bond of Phpr–Phpr (O1–H4) because the oxygen atom in Phpr could also act as a robust hydrogen bond acceptor. This could show that the hydrogen bond between Phpr and Phpr serves an essential role in the hydrogen bond network. As can be seen in Fig. 5a–d, the percent occupancy of the considered hydrogen bond, with the percent occupancy in the range of 80 to 100%, was decreased, as the temperature was raised. In other words, the strength of the hydrogen bond was reduced with the temperature increment.

### Interaction energies

**Figure 6:**
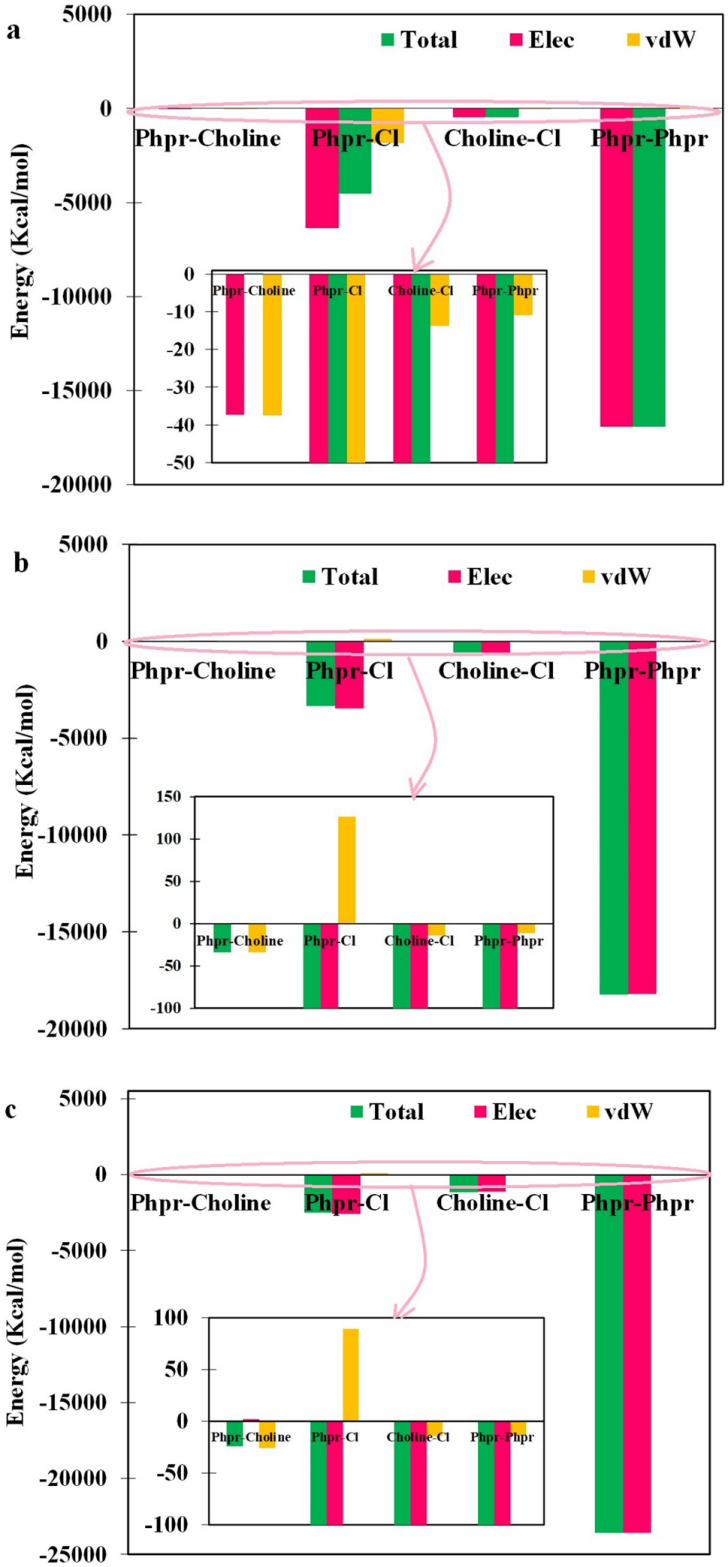
Interaction energies of Phpr-choline, Phpr–Cl^−^, choline–Cl^−^, Phpr–Phpr in DES were calculated using ‘NAMD energy’ plugins of the VMD package^40^ at (**a**) 293 K, (**b**) 321 K and (**c**) 400 K.

As we know, the DES melting point depends on the interaction energies of Phpr–Phpr, Phpr-cation, Phpr-anion, and cation–anion. The total non-bonded interaction energies, which were decomposed into electrostatic and van der Waals (vdW) components, were calculated using ‘NAMD energy’ plugins of the VMD package^40^ and reported at temperatures shown in Fig. 6. The interaction energies of Phpr–Phpr had a major contribution to the total intermolecular interaction energy, which was consistent with the results of RDF, hydrogen bond analyses. Furthermore, the interaction energies between Phpr molecules and chloride anions were larger than those between choline cation and Phpr molecules or chloride anions. In these systems, the van der Waals interaction had an insignificant contribution to the total intermolecular energy. The obtained results, as shown in Fig. 6, therefore, suggest that the total non-bonded interaction energies were increased with rasing temperature in the case of the interaction energy between Phpr and Phpr molecules and also, ion pairs (i.e., between chloride anion and choline cation.). However, the interaction energies of Cl^−^-Phpr, Phpr-choline were reduced when the temperature was raised. Additionally, it was found out that the attractive electrostatic interaction energy between DES species was observed for the studied temperatures. In the case of Phpr-Cl^−^, the vdW interaction energies were repulsive for 321, and 400 K, which helped to prevent the complexes’ aggregation. However, at the eutectic temperature, 293 K, all interactions were attractive.

### Dipole moment of HBD and HBA

Here, we compared the average values of the dipole moment of Phpr molecules and choline in the DES with another one in a pure state. This was calculated with the help of the MD simulations through fitting Eq. (1)^39^ to the distribution of the molecular dipole moments using the Gnuplot-5.2.6 fitting tool.

**Figure 7:**
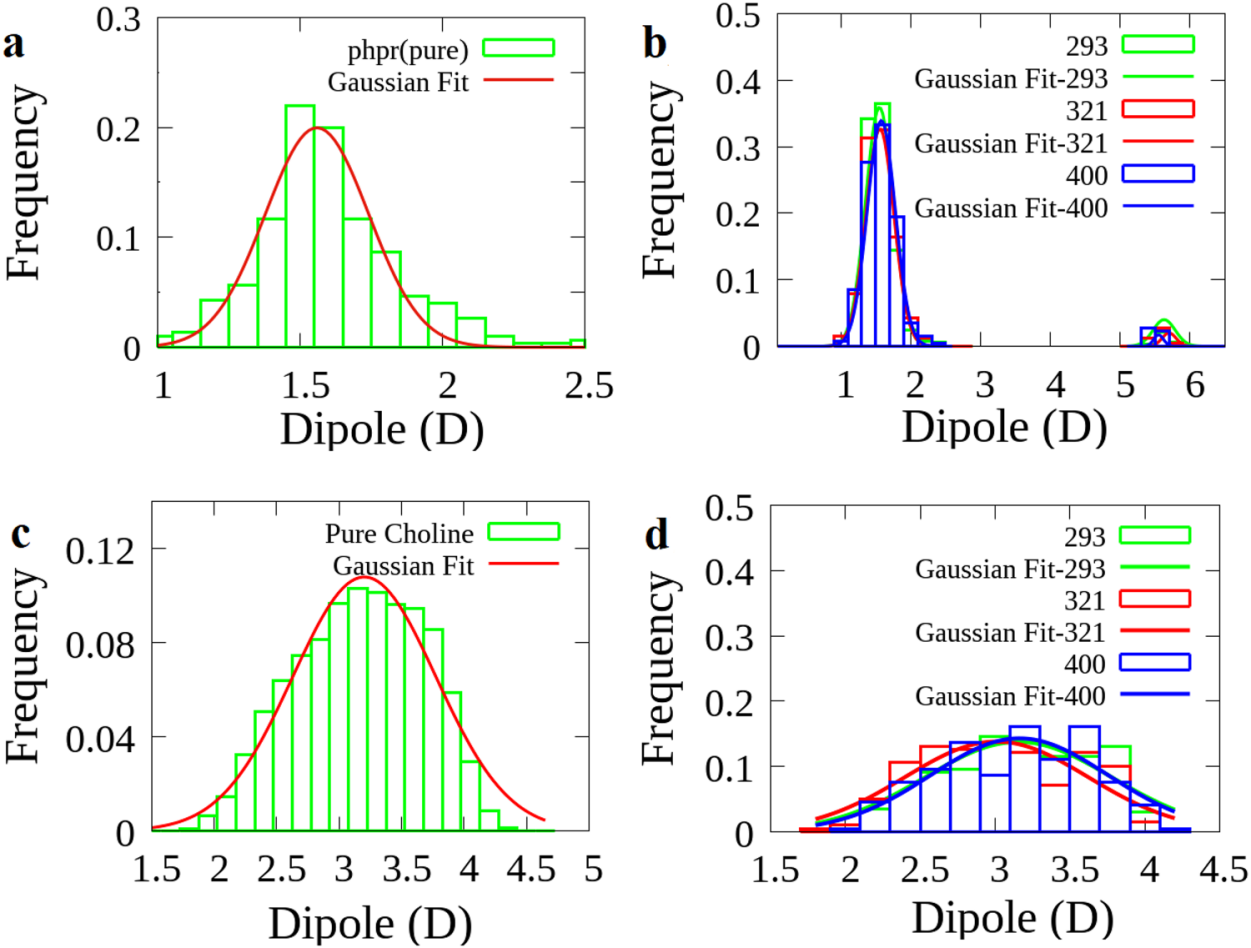
The normalized-distribution of molecular dipole moment vector of Phpr in a pure state; (**a**), in DES system; (**b**), and the normalized-distribution of choline cation in pure choline chloride system; (**c**) and in DES system; (**d**) The data were fitted using Gnuplot 5.2.6 (http://www.gnuplot.info/).

The distribution of the dipole moment values of choline cations and Phpr for the pure state as well as DES, is presented in Fig. 7a–d. The dipole moments of Phpr had an average value around 1.5 and 5.5 D, while this was around 1.5 D in the pure state. Therefore, the Phpr molecules had at least two distinct dipole moment distributions; consequently, two distinct average conformations were found in the studied DES. These distributions were observed at the three considered temperatures. Table 3 presents the average dipole moment values obtained from fitting along with the corresponding errors. The thermal-averaged dipole–dipole potential energy is expressed by the Keesom equation^41,42^. This equation has the following form

**Table 3:** The average dipole moment vector in Debye (D) and standard deviation of the distribution for the studied systems in a pure state and mixture at the considered temperatures in 0.1 MPa.

Average dipole moment (D)	Temperatures (K)			
	293 K	321 K	400 K	Pure systems
$\stackrel{-}{\mathrm{D}}$ of Choline	3.12 $\pm$ 0.08	3.01 $\pm$ 0.07	3.16 $\pm$ 0.08	3.20 ± 0.02
$\stackrel{-}{\mathrm{D}1}$ of Phpr	1.57 $\pm$ 0.01	1.58 $\pm$ 0.01	1.64 $\pm$ 0.01	1.56 ± 0.01
$\stackrel{-}{\mathrm{D}2}$ of Phpr	5.63 $\pm$ 0.2	5.58 $\pm$ 0.2	5.53 $\pm$ 0.2	–

With respect to the fact that the normalized distribution of dipole moment of Phpr have two Gaussian-typ behavior, therefore, two average dipole moment were reported ($\stackrel{-}{\mathrm{D}1}$ and $\stackrel{-}{\mathrm{D}2}$).

2 $$\langle \mathrm{V }(\mathrm{r})\rangle = \frac{-1}{3}\frac{{\upmu }_{\mathrm{A}}^{2}{\upmu }_{\mathrm{B}}^{2}}{{(4\mathrm{\pi \kappa }{\upvarepsilon }_{0 })}^{2}}\frac{1}{{\mathrm{r}}^{6}}\frac{1}{{\mathrm{K}}_{\mathrm{B}}\mathrm{T}},$$

where μ, r, and T are the dipole moment, distance and temperature, respectively^41^. Other symbols have their custom meanings. Ion–dipole interactions average^43^ is expressed by the following equation, which is very similar to the Kessom equation except for the power of *r*.

3 $$\langle \mathrm{V }(\mathrm{r})\rangle = \frac{-1}{3}\frac{{\mathrm{q}}^{2} {\upmu }^{2}}{{(4\mathrm{\pi \kappa }{\upvarepsilon }_{0 })}^{2}}\frac{1}{{\mathrm{r}}^{4}}\frac{1}{{\mathrm{K}}_{\mathrm{B}}\mathrm{T}}$$

According to the preceding section, in regared to RDFs, it was shown that the distances of Phpr, Cl^−^, and choline cation around Phpr remained almost unchanged with changing temperatures; therefore, the molecular dipole moment (μ) and temperature (T) serve the dominant roles in the variations of the dipole–dipole and ion–dipole potentials. So, by substituting the measured values for the molecular dipole moments of species and the corresponding temperatures, as given in Table 3, according to Eqs. (2) and (3), V(r) is the maximum value at the eutectic temperature. Therefore, the attractive dipole–dipole and ion–dipole potential energy, which is at the maximum value with the cooperation of Phpr–Cl^−^ and Phpr–Phpr hydrogen bonds network may lead to the depression of the melting point; most importantly, the one-component-like behavior of eutectic composition was observed. One, therefore, can say that the eutectic behavior exhibition is a result of some good balance between the values of molecular dipole moments, temperature and hydrogen bonds network between species.

**Figure 8:**
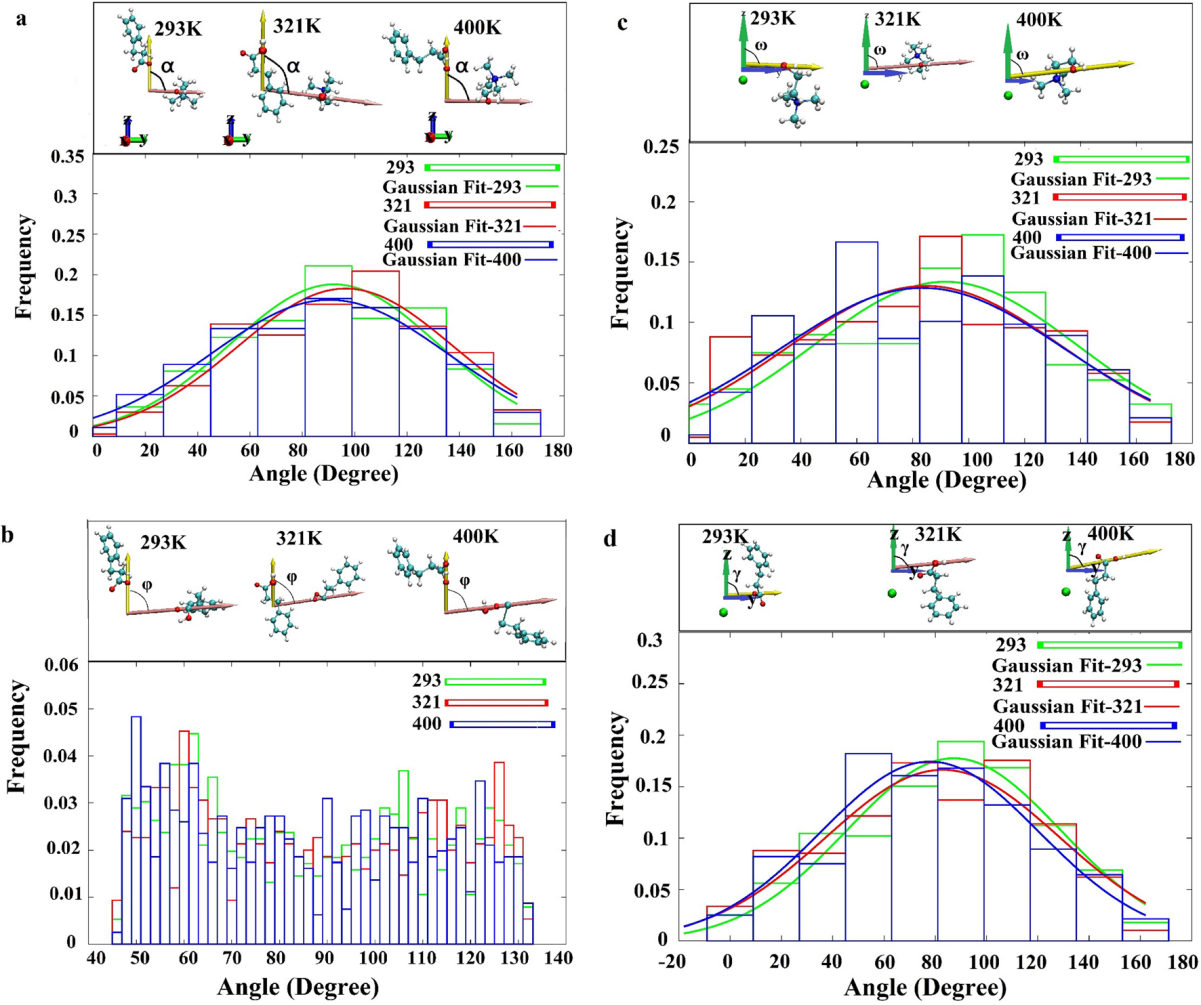
The normalized-distribution of the (**a**) angle of choline on Phpr, (**b**) Phpr on the Phpr, (**c**) choline on the chloride and (**d**) Phpr on the chloride at 293, 321 and 400 K in 0.1 MPa. The data were fitted using Gnuplot 5.2.6 (http://www.gnuplot.info/).

Based on a close examination of the results presented in the previous sections (RDFs, SDFs and hydrogen bonds), we focused on the angles between the dipole moment of the bonds as they could play an important role in the H-bond network. The orientation of the O-H4 bond dipole moment of Phpr around the dipole moment of H14-O1 bond’s choline cation (α) and C-O1 bond’s Phpr (β) was calculated, as well as the normalized distribution was depicted in Fig. 8a and b. The orientation of choline cation and Phpr around spherical chloride ions was also displaed in Fig. 8c and d. These figures show the average values of the angle of the choline’s H14–O1 bond dipole moment, and O–H4 bond’s Phpr with the z-axis, named ω and γ, respectively. These figures also indicate that the angles between O–H4 and C–O1 bonds of Phpr was distributed over 40°–130°, with the lowest population within the 70°–100° region. This was probably due to the formation of both inter and intramolecular hydrogen bonds in Phpr; the extent of intramolecular H-bonds was greater than that of the intermolecular H-bond. This would lead to reducing the interactions between dipole moments of the involved bonds. The results of fitting the Gaussian distribution to the measured angles are summarized in Table 4. It could be seen that the distribution of angles between the considered dipole moments was not dependent on the temperature.

**Table 4:** The average angle values between dipole moments of the bonds involved in the interactions.

Average angles (°)	Temperature (K)		
	293 K	321 K	400 K
(O1–H4) with (O1–H14) (⍺)	92.12 $\pm$ 3.4	96.54 $\pm$ 3.6	90.37 $\pm$ 2.5
(O1–H14) with z-axis (ω)	91.7 ± 5.7	84.56 ± 5.7	83.45 ± 7.1
(O1–H4) with z-axis (γ)	87.24 ± 2.1	82.93 ± 4.4	77.90 ± 3.1

### Density

Density is one of the properties that can be considered to determine the extent of molecular interactions, development of the equation of states, and the effect of pressure on the thermodynamical properties of the pure and mixed materials. A survey of the related literature showed that the density of DES at 25 °C was between 1 and 1.35 g cm^−3^^5,44^. The densities of ChCl/Phpr with a 67 mol% Phpr, fell in the range of 1.004–1.09 g cm^−3^ for the temperatures of 293, 321, and 400 K. All the related data were summarized in Table 5. Our finding suggests the densities were decreased with raising temperature, which was consistent with the results obtained by of Mjalli groups^44^.

**Table 5:** The densities and viscosities of deep eutectic solvents at 293, 321and 400 K.

Temperature (K)	293 K	321 K	400 K
Density (g cm^−3^)	1.081	1.057	1.004
Viscosity (mPa s)	308.674	207.282	20.520

Abbot et al.^12^ also indicated that the density of acetamide/ZnCl_2_ and urea/ZnCl_2_ eutectic mixtures was dependent on the packing and molecular organization. The densities of the studied ChCl/Phpr system were declined with increasing the temperature, thus suggesting the growth of hole radius or vacancy in the system, which caused an increase in the mass transport properties.

### Self-diffusion coefficient

To characterize the microscopic dynamics of deep eutectic solvents, the self-diffusion coefficient given by the Einstein's relation was analysed.

4 $$\begin{array}{c}\begin{array}{c}D=\underset{\mathrm{t}\to \infty }{\mathrm{lim}}{\frac{1}{6\mathrm{t}}<|{\mathrm{r}}_{\mathrm{i}}\left(\mathrm{t}\right)-{\mathrm{r}}_{\mathrm{i}}\left(0\right) |}^{2}>\end{array}\end{array}$$

In this equation, $\mathrm{D}$ is the diffusion coefficient, r_i(t)_ represents the position of the particle i at time t, and t = 0. The 〈|r_i_(t)-r_i_(0)|^2^〉 is the mean square displacement (MSD) of the particle i. The angle brackets <> indicate the average over time origins^45,46^.

The self-diffusion coefficient was obtained in the region where MSD was linear in time; in other words, where the motion of particles was uncorrelated. It could be said that the system in this time region had a diffusive regime. This was obtained in a relatively long simulation time. It should be noted that, in a short MD simulation, Eq. (4) cannot be used for DESs^27,32,45–47^.

To determine the diffusive regime, the beta-parameter, $\upbeta$, was calculated by following Del Popol^42^ and using Eq. (5):

5 $$\upbeta \left(\mathrm{t}\right)=\frac{\mathrm{dlog}\langle \mathrm{\Delta r}{\left(\mathrm{t}\right)}^{2}\rangle }{\mathrm{dlogt}},$$

where $\mathrm{\Delta r}{\left(\mathrm{t}\right)}^{2}$ and *t* indicate the MSD of the species and time, respectively. For both $\upbeta \hspace{0.17em}$< 1 and $\upbeta >1$, the system is in the sub-diffusive regime; in  $\upbeta$ = 1, the system is in the diffusive regime. Thus, the self-diffusion coefficient was calculated when $\upbeta$ parameters approached unity.

**Figure 9:**
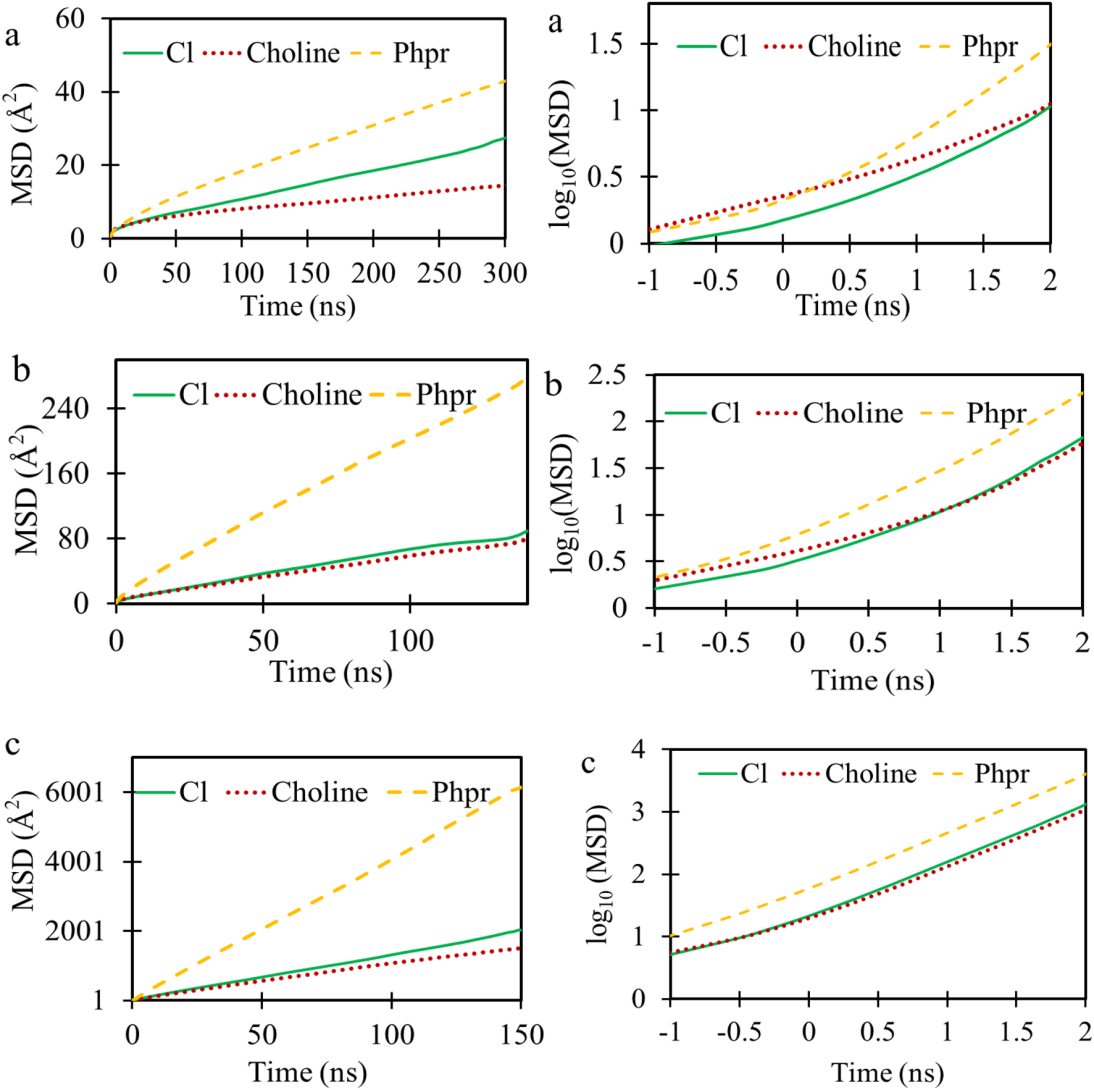
The linear (left panel) and logarithm scale (right panel) of the center of mass MSD for species were calculated using the VMD package^40^ at the temperatures; 293 K: (**a**), 321 K: (**b**) and 400 K: (**c**).

**Figure 10:**
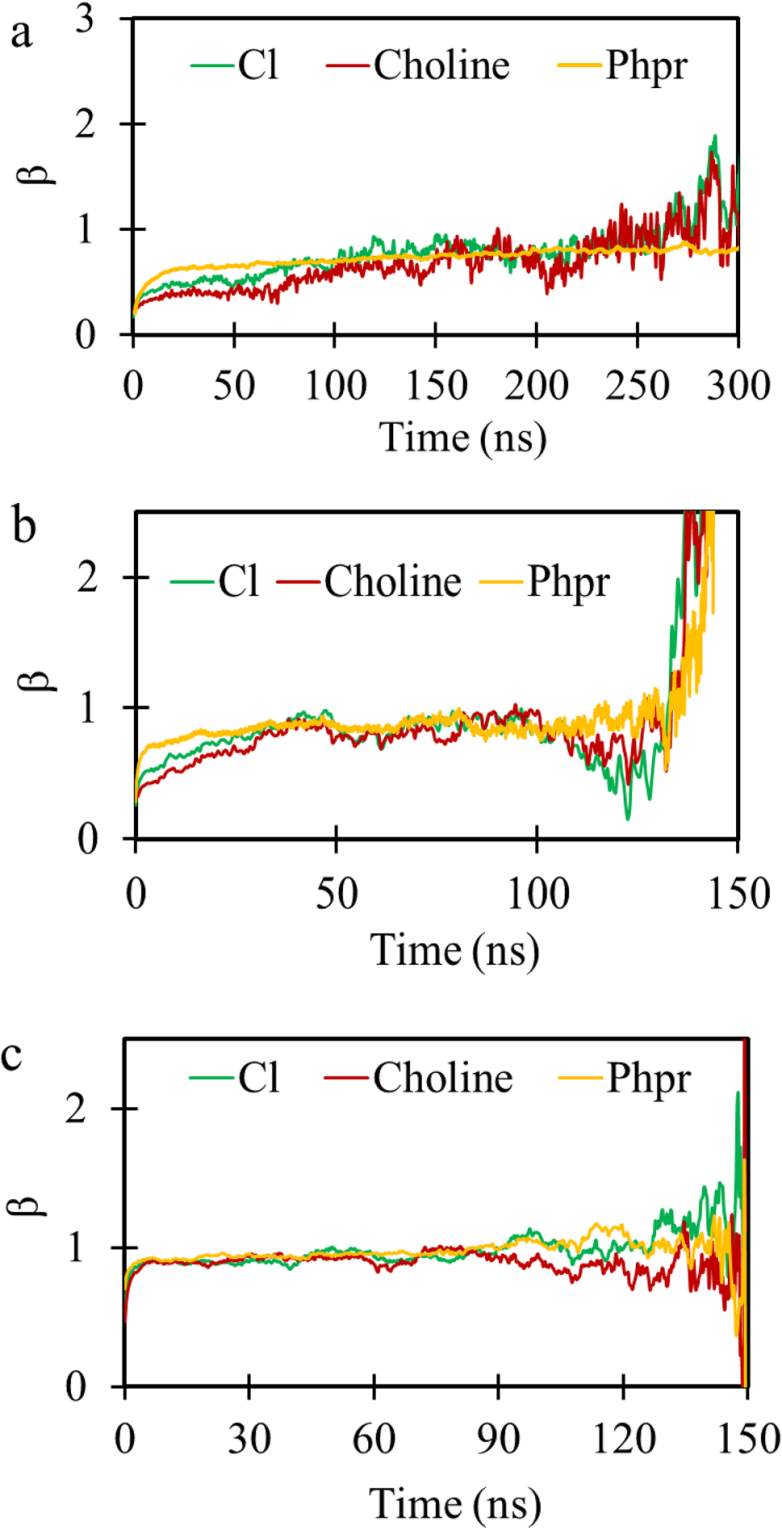
The variation of β with time for chloride (green), choline (red) and phenyl propionic acid (yellow) in DES were plotted by Gnuplot 5.2.6 (http://www.gnuplot.info/) at 293: (**a**); 321: (**b**); 400; (**c**) and in 0.1 MPa.

Figure 9 shows the MSDs of chloride, choline, and Phpr molecules on linear and logarithmic scales for DES contained 600 molecules at considered temperatures (293, 321, and 400 K). To calculate the self-diffusion coefficient of the DES species from MD simulations, the β parameter was evaluated using Eq. (5); and its variations with the simulation time at considered temperatures was shown in Fig. 10. Clearly, the required time for approaching a diffusive regime was reduced as the temperature was increased.

Given that, the self-diffusion coefficient was affected by the system size, the obtained self-diffusion coefficient of DES species from MD simulations (Eq. 4) was corrected using the Yeh-Hummer correction, ${\mathrm{D}}^{\mathrm{YH}}$^48–51^,

6 $${\mathrm{D}}_{\mathrm{i},\mathrm{ self}}^{\infty }= {\mathrm{D}}_{\mathrm{i},\mathrm{ self}}^{\mathrm{MD}}+ {\mathrm{D}}^{\mathrm{YH}}= {\mathrm{D}}_{\mathrm{i},\mathrm{ self}}^{\mathrm{MD}}+ \frac{{\mathrm{k}}_{\mathrm{B}}\mathrm{ T \xi }}{6\mathrm{ \pi \eta L}},$$

where ${\mathrm{D}}_{\mathrm{i},\mathrm{ self}}^{\infty }$ and ${\mathrm{D}}_{\mathrm{i},\mathrm{ self}}^{\mathrm{MD}}$ are the self-diffusion coefficients for the infinite (thermodynamics limit) and finite-size system, respectively. Also, η, T, ${\mathrm{k}}_{\mathrm{B}}$ and L are viscosity, temperature, constant Boltzmann, and length of the simulation box, respectively. ξ is a constant equal to 2.837298 for a cubic simulation box. The self-diffusion coefficient of a molecule in the thermodynamic limit (${\mathrm{D}}_{\mathrm{i},\mathrm{ self}}^{\infty }$) can be calculated by the extrapolation of Eq. (6) to 1/L → 0.

**Figure 11:**
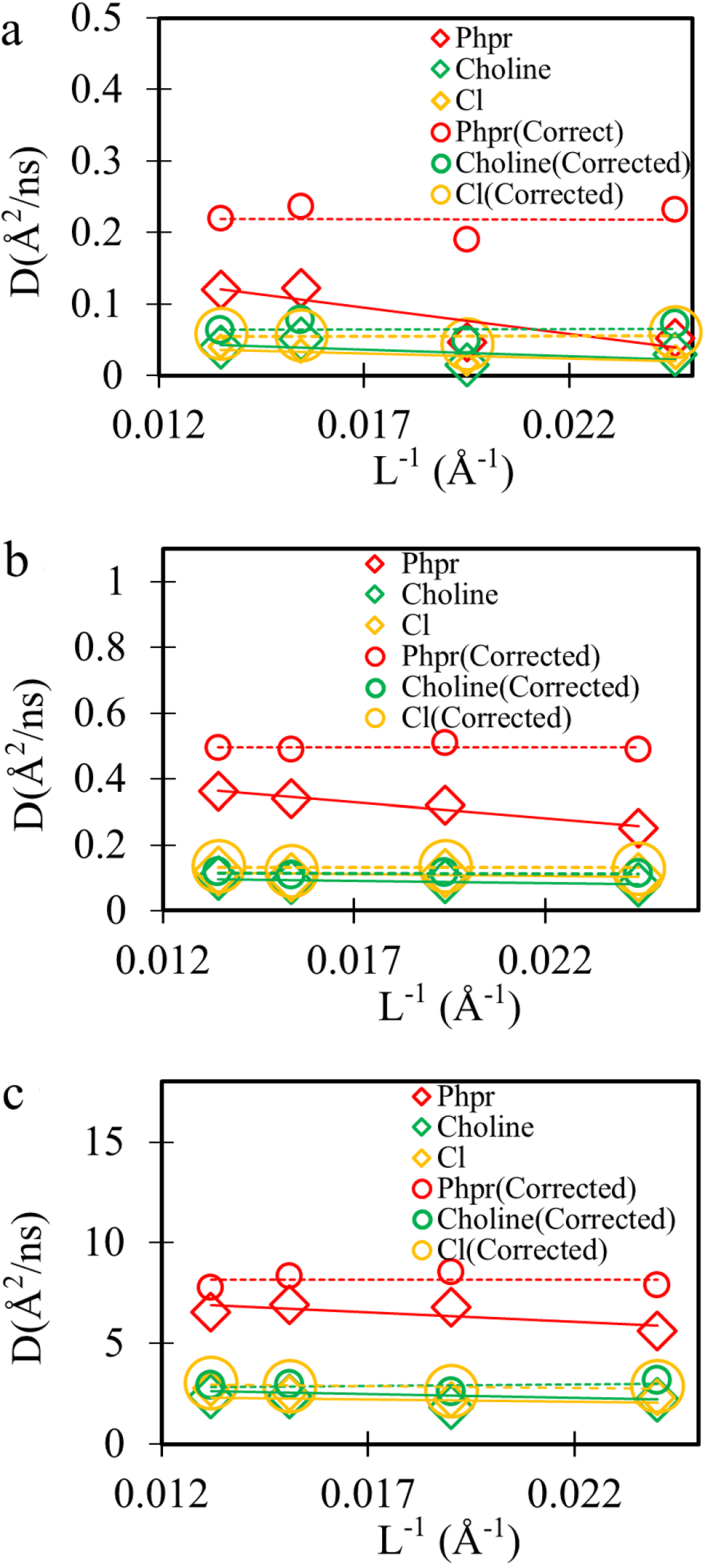
The self-diffusion coefficient of DES species as a function of invers box length at (**a**) 293 K, (**b**) 321 K, and (**c**) 400 K. The uncorrect self-diffusion coefficient (${\mathrm{D}}_{\mathrm{i},\mathrm{ self}}^{\mathrm{MD}}$) from MD simulations are depicted as diamonds, and the solid lines are the extrapolation to the thermodynamic limit (${\mathrm{D}}_{\mathrm{i},\mathrm{ self}}^{\infty }$). The corrected self-diffusion coefficient using Eq. (6) are shown as circles, and the dash lines are the extrapolated values. These plots were drawn by Gnuplot 5.2.6 (http://www.gnuplot.info/).

The system size effects on MSD of DES species for systems ranging from 300 to 1800 molecules were also displayed in Fig. S1 of the Supporting Information, indicating the slope of the MSD of DES species depends on the system size. The self-diffusion coefficient of DES species from MD simulations (${\mathrm{D}}_{\mathrm{i},\mathrm{ self}}^{\mathrm{MD}}$), as well as the self-diffusion coefficient of DES species in the thermodynamic limit (${\mathrm{D}}_{\mathrm{i},\mathrm{ self}}^{\infty })$ for the system ranging from 300 to 1800 molecules at 293, 321, and 400 K were listed in Table 6. As seen in Fig. 11, the calculated self-diffusion coefficient of DES species from MD simulations as a function of system size varies linearly with the inverse of the box length at considered temperatures. However, the corrected self-diffusion coefficient using Eq. (6) on the horizontal line indicted the validity of this correction method^49^.

**Table 6:** The self- diffusion coefficient (Å^2^/ns) of chloride anion, choline cation and phenyl propionic acid molecules (Phpr) obtaine from MD simulations for different size DES system and thermodynamic limit (${\mathrm{D}}_{\mathrm{i},\mathrm{ self}}^{\infty }$) at 293, 321 and 400 K and 0.1 MPa.

Size	293 K	321 K	400 K
**D**_**Phpr**_** (Å**^**2**^**/ns)**			
300	0.052 ± 0.001	0.251 ± 0.002	5.64 ± 0.04
600	0.047 ± 0.002	0.320 ± 0.003	6.79 ± 0.02
1200	0.123 ± 0.002	0.340 ± 0.002	6.924 ± 0.0009
1800	0.120 ± 0.003	0.364 ± 0.002	6.56 ± 0.01
${\mathrm{D}}_{\mathrm{i},\mathrm{ self}}^{\infty }$	0.220	0.497	8.146
**D**_**Choline**_** (Å**^**2**^**/ns)**			
300	0.030 ± 0.001	0.08 ± 0.001	2.22 ± 0.02
600	0.015 ± 0.002	0.090 ± 0.001	1.826 ± 0.01
1200	0.051 ± 0.001	0.087 ± 0.001	2.353 ± 0.02
1800	0.040 ± 0.002	0.099 ± 0.001	2.38 ± 0.005
${\mathrm{D}}_{\mathrm{i},\mathrm{ self}}^{\infty }$	0.066	0.114	3.165
**D**_**Cl**_^**−**^** (Å**^**2**^**/ns)**			
300	0.026 ± 0.001	0.101 ± 0.002	2.33 ± 0.02
600	0.017 ± 0.002	0.1140 ± 0.002	2.216 ± 0.008
1200	0.035 ± 0.001	0.1027 ± 0.001	2.54 ± 0.01
1800	0.040 ± 0.002	0.121 ± 0.0009	2.76 ± 0.007
${\mathrm{D}}_{\mathrm{i},\mathrm{ self}}^{\infty }$	0.056	0.1309	2.604

The results revealed that the corrected self-diffusion coefficient of the DES species was raised as the temperature was increased. This behavior implied that the H-bond network was diminished by rise of temperature. The dat, as presented in Table 6, showed that, chloride and choline ions moved more slowly than Phpr at the considered temperatures. Despite strong H-bonds between Phpr and Cl^−^, the corresponding diffusion coefficient of Phpr was higher than that of chloride. Therefore, their mobilities were not correlated.

**Figure 12:**
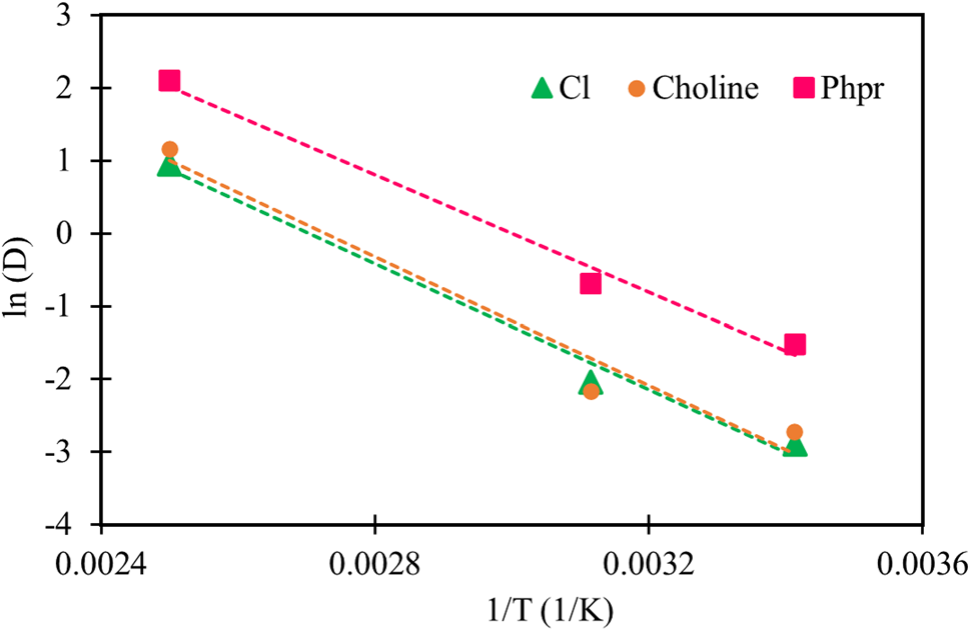
Correlation between the logarithm and the reciprocal temperature (Arrhenius plot) for the diffusion coefficients of the Cl^−^ (green), choline (orange) and HBD, Phpr, (pink) in investigated DES. Data plots were generated by gnuplot 5.2.6 (http://www.gnuplot.info/).

The correlation between the logarithm of the corrected self- diffusion coefficients of the constituents of DES and the reciprocal temperature is indicated in Fig. 12. This figure shows that the temperature dependence of the self-diffusion coefficients can be expressed by an Arrhenius-type equation:

7 $$ {\text{lnD}} = {\text{A}} + {{{\text{E}}_{{\text{a}}} } \mathord{\left/ {\vphantom {{{\text{E}}_{{\text{a}}} } {{\text{RT}}}}} \right. \kern-\nulldelimiterspace} {{\text{RT}}}}, $$

where A is the intercept of the equation (such as frequency or pre-exponential factor in the Arrhenius equation), and the slope of the equation (E_a_/R) can be interpreted as the activation energy of the diffusion process^44,52^. The obtained pre-exponential factor and activation energy for the diffusion were presented in Table 7.

**Table 7:** The Arrhenius equation parameters of species of studied DES.

Species	Chloride	Choline	Phpr
**Parameters of the Arrhenius equation**			
E_a_/R	− 4310.6	− 4418.7	− 4046.2
A	− 11.651	− 12.05	− 12.137

### Viscosity

Viscosity is a transport property serving an important role in the design, control, and optimization of the processes, including fluids such as DESs. Therefore, here, we calculated this quantity for the studied systems. The determination of the viscosity of DESs by MD simulation is a challenging task, mainly due to the high viscosity of DESs^20,53^. Viscosity is calculated by two common methods in the MD simulations. These are the Green–Kubo (GK) formalism and the Stocks-Einstein formula, which are applied in the equilibrium and non-equilibrium MD, respectively.

In this paper, we used the Green–Kubo relation^54,55^ for viscosity, as given by:

8 $$\upeta=\frac{\mathrm{V}}{3{\mathrm{k}}_{\mathrm{B}}\mathrm{T}}{\int }_{0}^{\infty }\left<{\sum }_{{\upalpha }<\upbeta }{\mathrm{P}\left(0\right)}_{{\upalpha }}\cdot {\mathrm{P}\left(\mathrm{t}\right)}_{{\upalpha \upbeta }}\right>\mathrm{dt},$$

where V, P_α β_, T, t and ${\mathrm{k}}_{\mathrm{B}}$ refer to volume, α, β components of pressure tensor (α, β = x, y, z), temperature, time and Boltzmann constant, respectively.

Viscosity is calculated using a home-made FORTRAN code, VISCO-MSL. It should be noted that the Green–Kubo method has been applied in the VISCO-MSL code to calculate the viscosity. This code has been validated by calculating of the viscosity of water at 298.15 K. The calculated viscosity of water had 10.53% relative error with the experimental value of 0.892 mPa s taken from Harris’ group^56^.

In order to calculate the viscosity of the considered DESs, the *β*-values obtained in the previous section were used. Table 5 summarizes the calculated viscosity values for the studied systems at the considered temperatures. These results showed that the obtained viscosities were decreased as the temperature was increased, as expected from the experimental results^36^. The maximum value for the viscosity was obtained at the temperature of 293 K. This was probably due to the enhanced Phpr–Phpr, Phpr–Cl^−^ and choline-choline electrostatic interactions at the eutectic temperatures, as discussed in the non-bonded interaction section.

It should be noted that the obtained viscosity of the studied DES at 321 was in a good agreement with the experimental data reported by the Abbot’s group^36^, showing a13.404% error.

## Materials and methods

**Figure 13:**
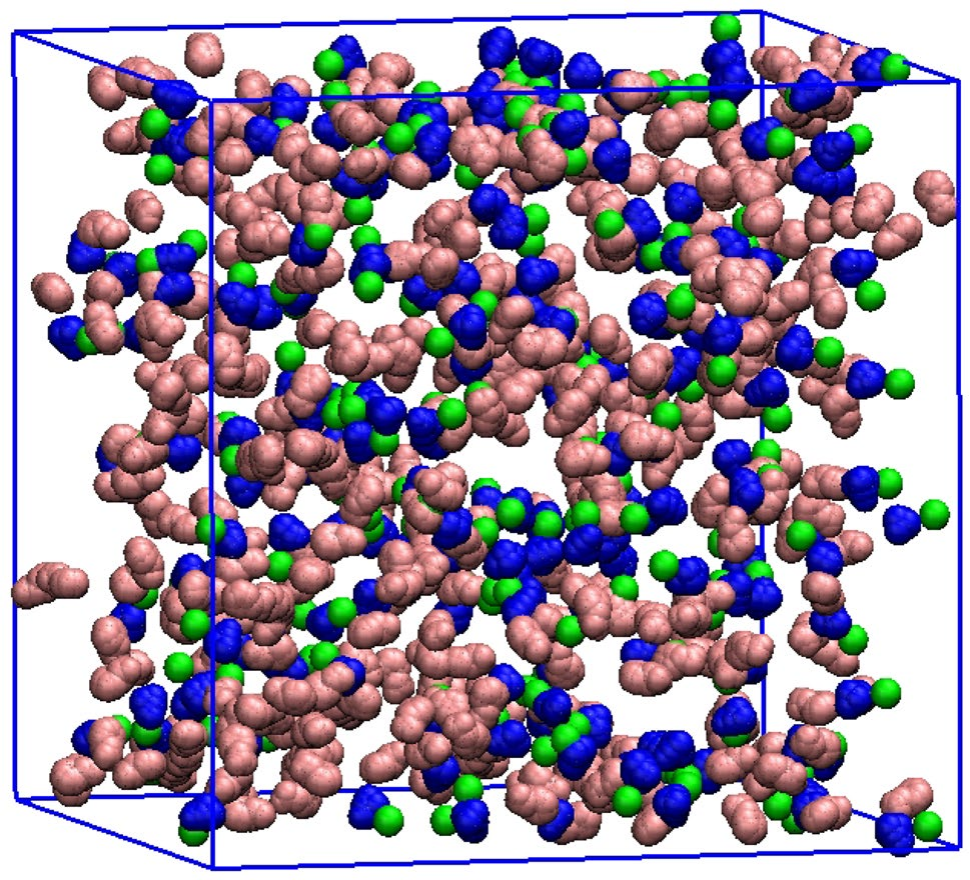
The initial configuration of the studied DES containing 402 Phpr; pink color, 198 choline; blue color, and 198 Cl^−^; green color was randomly generated using the PACKMOL package^57^.

Molecular dynamics (MD) simulations for the considered DES were carried out using cubic boxes consisted of 67 mol% phenyl propionic acid molecules (Phpr) and 33 mol% choline chloride (ChCl). The number of DES molecules and the average box lengths for the all systems were reported at 293, 321 and 400 K in Tables S1 and S2 of the Supporting Information, respectively. The initial configuration of the DES was randomly generated by the PACKMOL package^57^, as shown in Fig. 13. The CHARMM-36^58^ force field was chosen; the force field parameters were generated by the Swiss PARAM-Server^59^. It should be noted that the reduced-charge model is used to improve the prediction of transport and bulk-phase properties are used for DESs and ILs^31,32^. Therefore, the partial charges of all atoms in choline chloride, as obtained from Pekins et al.^31,32^, were scaled by a constant factor of 0.8 e in the studied DESs. Additionally, the partial charges of phenyl propionic acid were obtained using the restrained electrostatic potential charge (RESP) derivation method^60^, as provided by R.E.D-Tools^61–63^. Also, all Lennard–Jones (L–J) parameters and partial charges are listed in Table S3 and S4 of the Supporting Information.

NAMD package^64^ was used for all simulations. Nose–Hoover Langevin barostat^65^ with an oscillation period of 100 fs and a damping factor of 50 fs, was applied to control the system’s pressure. Langevin thermostat^66^, with a collision frequency of 5 ps^−1^, was also used to control temperature. Newton’s equations of motion with a time step of 1 fs were integrated for all simulations. The particle mesh Ewald (PME) method^67,68^, with a cutoff radius of 12 Å and grid spacing of 1 Å, was applied to evaluate the long-range intermolecular electrostatic interactions. Periodic boundary conditions (PBC) were also implemented in all three directions to remove the edge effect.

According to the protocol, First, the system was minimized; then each of the systems was heated from 0 K to 293, 321 and 400 K. They were equilibrated in the NVT ensemble for 5 ns. Subsequently, the production run of 150 ns was carried out in the isothermal-isobar ensemble, NPT, except T = 293. At 293 K; the simulation was performed for 300 ns under constant NPT conditions. All production runs were performed at 0.1 MPa.

Molecular graphics and simulation analyses such as RDFs, H-bond, percent occupancy, interaction energies, dipole moment of species, MSD and self-diffusion coefficient were performed using the Visual Molecular Dynamics program, VMD^40^. The SDFs of the species were calculated using TRAVIS^61^ software. The hydrogen bond and nonbond interactions analyses were carried out over a 20 ns trajectory. The RDF curves, SDFs, and molecular dipole moment of species were averaged over three simulation boxes during a long production run. Following long production runs in the NPT ensemble, the 50 ns run was performed in the NVT ensemble to evaluate the viscosity. To calculate the self-diffusion coefficient, the system size effect was investigated by performing simulations with the larger size. The MD simulations of these systems were performed using the same protocol mentioned above.

## Conclusion

Molecular dynamics simulations were performed on the choline chloride-based deep eutectic solvent with phenyl propionic acid (Phpr) as the HBD at the temperatures of 293, 321 and 400 K. The structural analyses, namely RDF and SDF, were then conducted to illustrate and confirm the formation of Phpr-choline, Phpr-Cl^−^, and Phpr–Phpr hydrogen bonds; the results were used to demonstrate the importance of Cl^−^–Phpr and Phpr–Phpr interactions in the formation of the hydrogen bond network. The calculated values of the molecular dipole moment vector of the species along the simulation did not change significantly with the variation of temperatures. However, according to the equations considered for the Keesom energy and ion-dipole interaction energy, the maximum attraction occurred at the eutectic temperature. So, this contributes to lowering the melting point in the eutectic temperature.

Dynamical properties such as self-diffusion coefficient and viscosity, as well as their relationship with temperatures, were calculated; these were in a good agreement with the experimental data. These results, therefore, suggested that at the eutectic temperature, the range of the variations of the self-diffusion coefficients of species, had a small width that became more extensive when the temperature was increased.

Finally, our predicted results from MD simulations can be applied in different areas such as the optimal design of solvent in various temperatures for specific industrial processes. Likewise, the results of this work can be used in the investigation of phase transition.

## Supplementary Information

Supplementary Information
