# Supplementary material for: Insights into the interactions and dynamics of a DES formed by phenyl propionic acid and choline chloride
Source: Sci Rep. 2021 Mar 18;11:6384. doi: 10.1038/s41598-021-85260-z (PMC7973556; doi:10.1038/s41598-021-85260-z)
Supplement: Supplementary file 1 — Supplementary Information [file 41598_2021_85260_MOESM1_ESM.pdf]

Insights into the Interactions and Dynamics of a DES Formed by Phenyl Propionic Acid and Choline Chloride

Parisa Jahanbakhsh Bonab[1, 2], Alireza Rastkar Ebrahimzadeh[2, 3], and Jaber Jahanbin Sardroodi[*, 1, 2]

[1] Department of Chemistry, Faculty of Basic Sciences, Azarbaijan Shahid Madani University, Tabriz, Iran.
[2] Molecular Simulation Laboratory (MSL), Azarbaijan Shahid Madani University, Tabriz, Iran.
[3] Department of Physics, Faculty of Basic Sciences, Azarbaijan Shahid Madani University, Tabriz, Iran.
[*] E-mail: jsardroodi@azaruniv.ac.ir

Table S1. The number of Phpr and ChCl molecules in each simulation box at 293, 321 and 400 K.

Number of DES molecules / DES species	300	600	1200	1800
**Phpr**	201	402	804	1206
**ChCl**	99	198	396	594

Table S2. The average lengths of simulation boxes at 293, 321 and 400 K.

Number of DES molecules / Temperature(K)	300	600	1200	1800
**293**	40.6847	40.6847	40.6847	40.6847
**321**	40.9321	40.9321	40.9321	40.9321
**400**	41.6675	41.6675	41.6675	41.6675

Table S3. The partial charges and force field vdW parameter for choline chloride, ChCl.

Atom type of ChCl	Partial charges of ChCl	ε_i (kcal/mol)	σ_i (Å)
C1, C2, C3	-0.10736	0.05500	4.35
H1, H2, H3, H4, H5, H6, H7, H8, H9	0.09544	0.02200	2.64
N1	0.04016	0.20000	3.7
C4	-0.02576	0.05500	4.35
H10, H11	0.08928	0.022000	2.64
C5	0.12008	0.05500	4.35
H12, H13	0.0408	0.02200	2.64
O1	-0.49512	0.152100	3.54
H14	0.3636	0.046000	0.449
Cl1	-0.8	0.1500	4.54

Table S4. The partial charges and force field vdW parameters for phenyl propionic acid, Phpr.

Atom type of Phpr	Partial charges of Phpr	ε_i (kcal/mol)	σ_i (Å)
C	0.7799	0.1100	4.00
C1	0.0079	0.05500	4.35
C2	-0.1212	0.05500	4.35
C3	0.0418	0.0700	3.9848
C4	-0.1212	0.0700	3.9848
C5, C7	-0.1905	0.0700	3.9848
C6	-0.0818	0.0700	3.9848
C8	-0.1212	0.0700	3.9848
H, H1	0.0335	0.02200	2.64
H2, H3	0.0568	0.022000	2.64
H4	0.4669	0.04600	0.449
H5	0.1236	0.02200	2.64

Figures

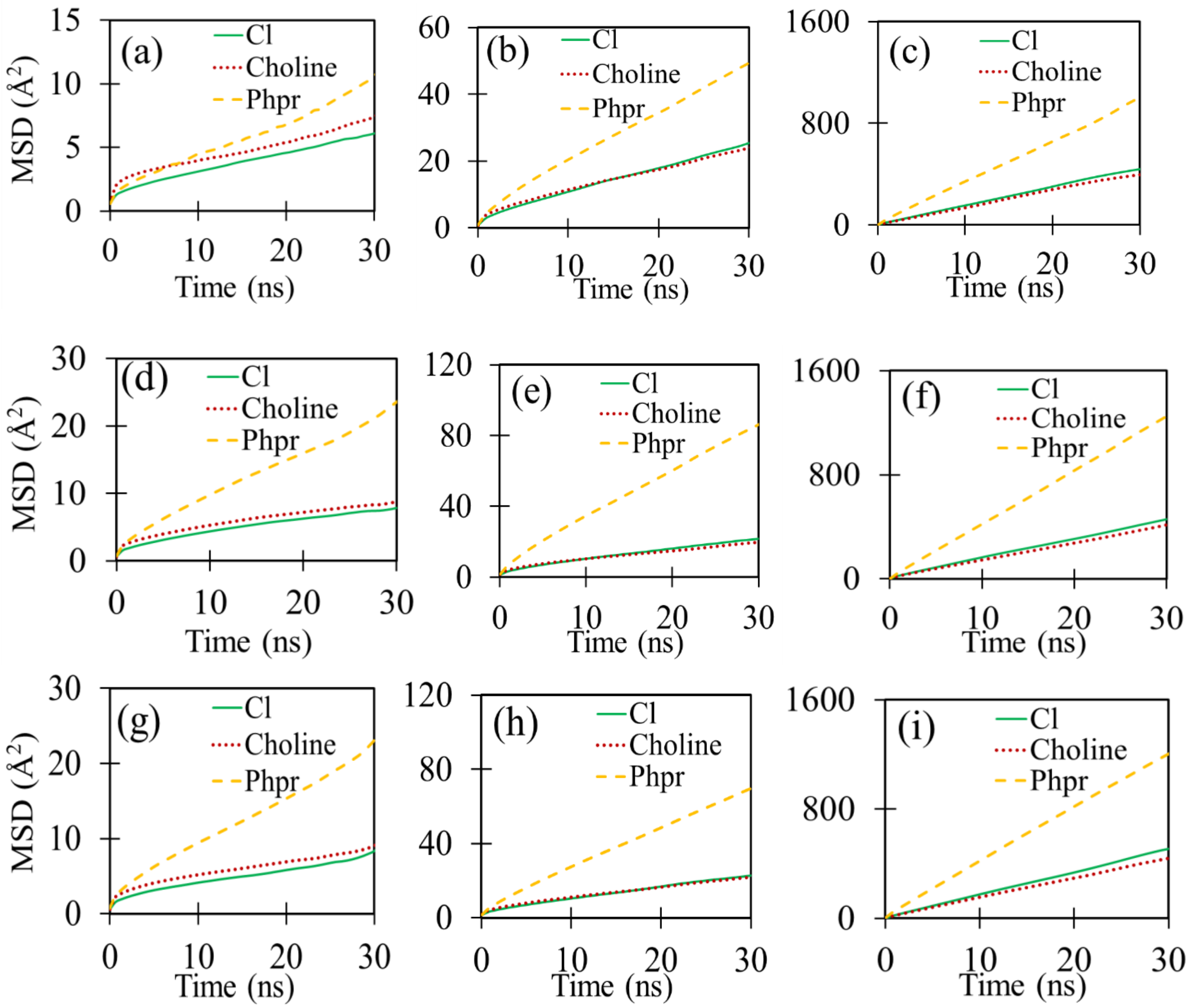

Figure S1. The comparison of mean square displacement (MSD) of DES species were calculated using the VMD package (1) for 300 DES molecules; a-c, 1200 DES molecules; d-f and 1800 DES molecules; g-I at 293 K (left panel), 300 K (middle panel) and 400 K (right panel).

Reference

1. Humphrey, W., Dalke, A. & Schulten, K. VMD: visual molecular dynamics. J. Mol. Graph. 14, 33–38 (1996).
